# Prevalence of fruite (kiwi) allergy in health worker with latex hypersensitivity

**DOI:** 10.1186/2045-7022-1-S1-P33

**Published:** 2011-08-12

**Authors:** Sayyed Hesamedin Nabavizadeh, Shiva Yazdanpanah

**Affiliations:** 1 Yasouj University of Medicine, Allergy, Yasouj, Islamic Republic of Iran; 2 Valfajr Health Center, Health Center, Shiraz, Islamic Republic of Iran

## 

Allrgic reaction to natural rubber latex have increased during past 10 years especially among health worker and patients with high exposure to latex allergen. Latex allergy is associsted with clinical or serological cross reactivity to plant derived food allergcn especially tropical fruit for example avocado bannana chestnut kiwi papaya potato and peaches. In this study on health worker among 580 participants 104(17.9 %) who were positive to latex skin prick test.Of 464 patients with negative skin prick test to latex are have 12 patiets with positive skin prick test to kiwi and in 197 patiens with positive skin prick test to latex 7 patients had positive skin prick test to kiwi( p<0.05). In this study difference of sensivity to banana and potato in both groups were not significant according to this study kiwi hypersensivity is important problem among health worker with sensivity to latex.

## Key words

latex Allergy -kiwi – skin prick test

